# Hybrid Phy-X/PSD–Geant4 Assessment of Gamma and Neutron Shielding in Lead-Free HDPE Composites Reinforced with High-Z Oxides

**DOI:** 10.3390/polym18020179

**Published:** 2026-01-09

**Authors:** Ahmed Alharbi, Nassar N. Asemi, Hamed Alnagran

**Affiliations:** 1 Department of Physics, College of Science, Qassim University, Buraydah 51452, Saudi Arabia; hnjran@qu.edu.sa; 2 Department of Computer Science, College of Engineering and Information Technology, Onaizah Colleges, Qassim 56447, Saudi Arabia

**Keywords:** HDPE, lead-free shielding, Bi_2_O_3_, WO_3_, Gd_2_O_3_, radiation-shielding composites, Geant4, Phy-X/PSD, gamma attenuation, fast-neutron removal cross section

## Abstract

This study evaluates lead-free high-density polyethylene (HDPE) composites reinforced with high-Z oxides (Bi_2_O_3_, WO_3_, Gd_2_O_3_, TeO_2_, and a Bi_2_O_3_/WO_3_ hybrid) as lightweight materials for gamma-ray and fast-neutron shielding. A hybrid computational framework combining Phy-X/PSD with Geant4 Monte Carlo simulations was used to obtain key shielding parameters, including the linear and mass attenuation coefficients (*μ*, *μ*/*ρ*), half-value layer (HVL), mean free path (MFP), effective atomic number (Z_eff_), effective electron density (N_eff_), exposure and energy-absorption buildup factors (EBF, EABF), and fast-neutron removal cross section (Σ_R_). The incorporation of heavy oxides produced a pronounced improvement in gamma-ray attenuation, particularly at low energies, where the linear attenuation coefficient increased from below 1 cm^−1^ for neat HDPE to values exceeding 130–150 cm^−1^ for Bi- and W-rich composites. In the intermediate Compton-scattering region (≈0.3–1 MeV), all oxide-reinforced systems maintained a clear attenuation advantage, with *μ* values around 0.12–0.13 cm^−1^ compared with ≈0.07 cm^−1^ for pure HDPE. At higher photon energies, the dense composites continued to outperform the polymer matrix, yielding *μ* values of approximately 0.07–0.09 cm^−1^ versus ≈0.02 cm^−1^ for HDPE due to enhanced pair-production interactions. The Bi_2_O_3_/WO_3_ hybrid composite exhibited attenuation behavior comparable, and in some regions slightly exceeding, that of the single-oxide systems, indicating that mixed fillers can effectively balance density and shielding efficiency. Oxide addition significantly reduced exposure and energy-absorption buildup factors below 1 MeV, with a moderate increase at higher energies associated with secondary radiation processes. Fast-neutron removal cross sections were also modestly enhanced, with Gd_2_O_3_-containing composites showing the highest values due to the combined effects of hydrogen moderation and neutron capture. The close agreement between Phy-X/PSD and Geant4 results confirms the reliability of the dual-method approach. Overall, HDPE composites containing about 60 wt.% oxide filler offer a practical compromise between shielding performance, manufacturability, and environmental safety, making them promising candidates for medical, nuclear, and aerospace radiation-protection applications.

## 1. Introduction

Radiation shielding is essential in nuclear research and engineering to safeguard people, patients, and sensitive equipment from ionizing radiation [1,2]. Gamma rays, a form of ionizing radiation, pose particular challenges because their strong penetration demands dense, high-atomic-number (Z) materials for effective attenuation [3]. Lead has historically been considered the standard shielding material because of its high density (11.34 g/cm^3^) and atomic number (Z = 82). However, the increasing concern over lead toxicity and its detrimental environmental and health effects has restricted its use, especially in medical, industrial, and environmental applications [4]. Concrete remains widely used for its affordability and availability; however, it requires large thicknesses to achieve adequate attenuation, resulting in heavy, non-portable shielding structures.

The shortcomings of conventional shields have encouraged the development of innovative materials that combine strong attenuation performance with low toxicity, reduced weight, and mechanical flexibility. Glass and ceramic matrices reinforced with heavy oxides such as WO_3_, Bi_2_O_3_, and TeO_2_ have shown improved gamma-ray shielding capability, but their brittleness and limited mechanical strength constrain practical applications [5,6,7,8]. Similarly, concrete enhanced with barite or magnetite demonstrates higher shielding efficiency but remains unsuitable where lightweight or mobile designs are required [9].

Polymers, particularly high-density polyethylene (HDPE), present an attractive alternative because of their low density, processability, and ability to incorporate high-Z fillers [10]. HDPE is especially valuable in mixed radiation fields owing to its high hydrogen content, which moderates neutrons while enhancing photon attenuation when combined with heavy reinforcements [11]. This dual capability enables HDPE composites to serve as lightweight, multifunctional materials for both gamma- and neutron-radiation protection. Earlier studies examined PbO-filled HDPE composites that exhibited strong gamma-ray attenuation; however, the inherent toxicity of lead limited their practical applicability [12,13]. Subsequent research explored environmentally benign configurations, including recycled HDPE reinforced with alternative heavy-metal oxides and polymer-based layered composite systems [14].

Among environmentally benign candidates, Bi_2_O_3_ has emerged as one of the most efficient and non-toxic lead-free substitutes, owing to its high atomic number (Z = 83), chemical stability, and environmental safety [15,16,17]. Similarly, WO_3_, Gd_2_O_3_, and TeO_2_ have been recognized for their strong photon-interaction cross sections, thermal stability, and compatibility with polymer matrices [18,19,20,21]. Although these oxides have individually shown excellent attenuation capability, only a few studies have systematically compared their combined influence when incorporated into polymer composites. Moreover, hybrid analyses that integrate Monte Carlo (Geant4) simulations and semi-empirical (Phy-X/PSD) methods for HDPE-based oxide systems remain scarce—particularly across the wide photon-energy range covering photoelectric, Compton, and pair-production regions. This absence of integrated evaluation defines the knowledge gap addressed in the present work. In particular, no previous study has provided a unified, side-by-side assessment of Bi_2_O_3_, WO_3_, Gd_2_O_3_, TeO_2_, and their hybrid formulations within an HDPE matrix using a dual-method framework that spans the entire 0.015–15 MeV photon-energy range, which establishes the core novelty of the present investigation.

Building on this context, the present study systematically investigates six HDPE-based composites: pure HDPE (100 wt.%), HDPE + 60 wt.% Bi_2_O_3_, HDPE + 60 wt.% WO_3_, HDPE + 60 wt.% Gd_2_O_3_, HDPE + 60 wt.% TeO_2_, and a hybrid formulation containing HDPE + 30 wt.% Bi_2_O_3_ + 30 wt.% WO_3_. These compositions were designed to balance high-Z reinforcement with polymer flexibility, enabling effective gamma- and neutron-radiation attenuation while maintaining manufacturability and non-toxicity. The dual characteristics of HDPE—its lightweight nature and mechanical resilience—combined with the high-density oxides create composites that are safer to handle, easier to shape, and adaptable for both portable and fixed shielding applications. To quantitatively evaluate their performance, a hybrid computational framework was employed, coupling Phy-X/PSD for semi-empirical calculations of photon- and neutron-interaction parameters with Geant4 Monte Carlo simulations for detailed photon-transport modeling. This integrative approach bridges analytical and stochastic methods, providing a consistent basis for assessing how filler composition and photon energy affect attenuation behavior. The results support the development of sustainable, lead-free polymer-oxide shielding materials suitable for medical, nuclear, aerospace, and security applications.

## 2. Research Significance and Novelty

This study addresses a clear gap in the literature. It provides a unified, cross-validated comparison of several high-Z oxide fillers within an HDPE matrix, offering insight not available in previous shielding research. By integrating Phy-X/PSD with Geant4, the work combines complementary analytical and Monte Carlo approaches, strengthening the reliability of attenuation estimates across the entire 0.015–15 MeV energy range. The evaluation of the Bi_2_O_3_/WO_3_ hybrid composite adds particular novelty, as its potential to balance density, attenuation performance, and practical manufacturability has not been previously examined within a dual-method framework. Together, these elements define the significance of the present study and its contribution to the development of safer, more efficient, and lead-free radiation-shielding materials.

## 3. Materials and Methods

### 3.1. Materials and Composite Design

The investigated materials included pure HDPE and five HDPE-based composites reinforced with different high-Z oxide fillers: Bi_2_O_3_, WO_3_, Gd_2_O_3_, TeO_2_, and a Bi_2_O_3_/WO_3_ hybrid mixture (30/30 wt.%). Each composite was formulated with 40 wt.% HDPE and 60 wt.% total oxide reinforcement, except for the hybrid system, which incorporated 30 wt.% of each oxide. The selected loading range is consistent with experimentally investigated HDPE-based shielding composites, where oxide concentrations up to approximately 50 wt.% are commonly reported. For example, Bi-filled HDPE systems reported by Sheela et al. (2019) [22] and Almuqrin et al. (2022) [23] fall within this range and demonstrate that such compositions are practically achievable and suitable for producing homogeneous HDPE–oxide mixtures.

A 60 wt.% filler loading was selected as a near-upper practical level, consistent with previous experimental polymer-based shielding composites in which 60 wt.% heavy-oxide fillers were successfully fabricated [24]. This loading provides a substantial increase in composite density and photon-interaction probability while maintaining acceptable mechanical integrity and processability, as generally reported for polymer matrices approaching high filler fractions. Accordingly, the adopted weight fractions were chosen to reflect experimentally supported, fabricable polymer–oxide composites while enabling meaningful enhancements in gamma- and neutron-attenuation behavior.

The selected fillers were chosen to provide synergistic attenuation mechanisms across different photon-energy ranges, including photoelectric absorption at low energies, Compton scattering at intermediate energies, and pair production at high energies. The adopted theoretical densities of the constituent materials were 0.94 g·cm^−3^ for HDPE, 8.90 g·cm^−3^ for Bi_2_O_3_, 7.16 g·cm^−3^ for WO_3_, 7.41 g·cm^−3^ for Gd_2_O_3_, and 5.99 g·cm^−3^ for TeO_2_. Using these values, the composite densities were calculated according to the mixture rule [25] based on the specified weight fractions, and the resulting compositions and densities are summarized in Table 1. These defined densities were subsequently used as fixed input parameters in both Phy-X/PSD and Geant4 simulations to ensure consistency across the two computational approaches.

### 3.2. Theoretical Calculations

The linear attenuation coefficient (*μ*, cm^−1^) quantifies the probability per unit path length that a photon interacts with a material and was determined from the Beer–Lambert exponential attenuation law, expressed as $I=I_{0}e^{-\mu x}$, where *I*_0_ is the incident intensity, *I* is the transmitted intensity after passing through a material of thickness *x*, and *μ* is the linear attenuation coefficient [26]. The mass attenuation coefficient (*μ*/*ρ*), defined as *μ* normalized by material density, provides a density-independent measure of photon-absorption efficiency and allows direct comparison between materials of different compositions and compactness [27].

The half-value layer (HVL = ln(2)/*μ*) represents the thickness required to reduce the photon flux by 50%, while the mean free path (MFP = 1/*μ*) denotes the average distance traveled by photons between successive interactions [27]. The effective atomic number (Z_eff_) and effective electron density (N_eff_) were obtained from the total atomic and electronic cross-sections, describing the composite’s overall photon-interaction strength across different energy regions. Z_eff_ describes the weighted atomic number of the composite and exhibits a strong dependence on photon energy: it is highest in the photoelectric-absorption region, where the interaction cross section scales approximately as $Z^{4-5}$; it decreases in the Compton-scattering region, where photon interactions depend primarily on electron density; and it increases slightly again at multi-MeV energies due to pair-production contributions. $N_{\mathrm{eff}}$ (electrons·$g^{-1}$) quantifies the number of electrons available for photon interactions per unit mass and follows a similar energy-dependent trend [28].

For assessing the contribution of scattered photons, the exposure buildup factor (EBF) and the energy-absorption buildup factor (EABF) were included. The EBF quantifies the increase in photon exposure resulting from multiple scattering events within the material, whereas the EABF represents the buildup of absorbed dose arising from these secondary photons. Both quantities depend strongly on photon energy and penetration depth, expressed in mean free paths. They remain small at low photon energies where the photoelectric effect dominates, increase significantly in the Compton-scattering region where wide-angle scattering is most prevalent, and show moderate growth at higher energies due to secondary photons generated by pair-production interactions. In this study, EBF and EABF were evaluated according to the ANSI/ANS-6.4.3-1991 methodology as implemented in the Phy-X/PSD platform [29].

For neutron shielding, the fast-neutron macroscopic removal cross section ($\sum_{R}$, cm^−1^) represents the probability per unit path length that fast neutrons are removed from the uncollided beam through their first interaction within a material. $\sum_{R}$ values were evaluated using the Phy-X/PSD platform, which couples elemental mass removal cross sections with the composite’s composition and density. The platform requires elemental mass fractions *wᵢ*. The relation follows the standard mixture rule to obtain the overall fast-neutron removal cross section (FNRC) as expressed by Equations (1) and (2) [30]:

(1) $$\frac{\sum_{R}}{\rho }=\sum_{i}w_{i}{\left(\frac{\sum_{R}}{\rho }\right)}_{i}$$

(2) $$\sum_{R}=\rho \sum_{i}w_{i}{\left(\frac{\sum_{R}}{\rho }\right)}_{i}$$

where ${\left(\frac{\sum_{R}}{\rho }\right)}_{i}$ is the i-th element mass removal cross section for fast neutrons (cm^2^·g^−1^), ρ is the bulk composite density (g·cm^−3^), and $\sum_{R}$ is the composite macroscopic removal cross section (cm^−1^).

### 3.3. Phy-X/PSD Computations

The Phy-X/PSD platform [31] was used to compute *μ*, *μ*/*ρ*, HVL, MFP, Z_eff_, N_eff_, $\sum_{R}$, and the buildup factors. This semi-empirical tool integrates evaluated photon- and neutron-interaction data within its internal database. Input parameters included elemental weight fractions and the calculated composite densities from Table 1, assuming a homogeneous medium and a monoenergetic narrow-beam geometry. All photon-related quantities were evaluated across the 0.015–15 MeV range for consistent comparison with Monte Carlo photon results.

For neutrons, the fast-neutron macroscopic removal cross section (Σ_R_) was obtained using the ANSI/ANS-6.4.3 methodology implemented in Phy-X/PSD. In this context, “fast neutrons” refer approximately to the 0.5–10 MeV region for which elemental removal cross sections are tabulated. The reported *Σ_R_* value represents the standard spectrum-averaged fast-neutron removal coefficient used for comparative material-level evaluation in shielding studies.

### 3.4. Geant4 Simulations

The Geant4 toolset (version 11.0.2) [32], developed at CERN, was used to simulate photon transport and interaction in the prepared composites. This object-oriented Monte Carlo framework, implemented in C++, allows a precise definition of material composition, source characteristics, physical interaction models, and detector geometry.

**Figure 1:**
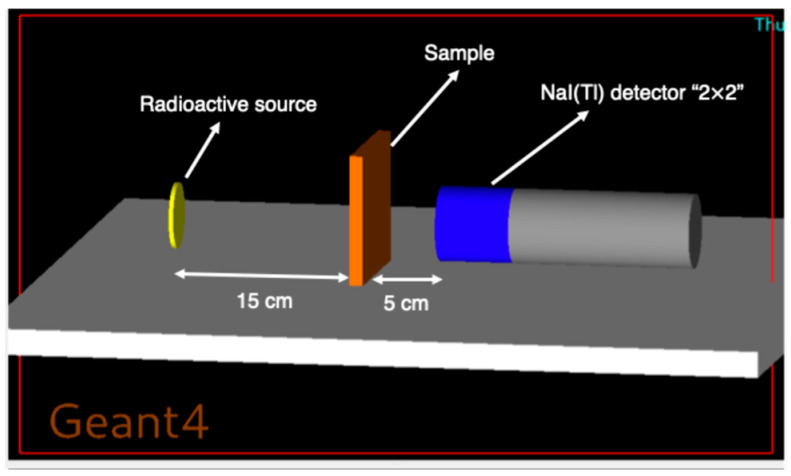
Geant4 setup for gamma-ray attenuation. A radioactive source (yellow) emits photons through the HDPE sample (orange) to a NaI(Tl) detector (2″ × 2″, blue/gray).

A monoenergetic, collimated photon beam (15 keV–15 MeV) was directed perpendicularly onto each composite slab, with a virtual detector positioned downstream to record transmitted flux. The selected physics list included photoelectric absorption, Compton scattering, pair production, and all secondary-particle transport processes, enabling an accurate description of photon propagation, energy deposition, and secondary-radiation generation in high-Z-reinforced polymers. Figure 1 illustrates the Geant4 setup for gamma-ray attenuation: a radioactive source emits photons through the HDPE sample toward a NaI(Tl) detector.

## 4. Results and Discussion

### 4.1. Gamma-Ray Attenuation Behavior (Linear Attenuation Coefficient, μ)

**Figure 2:**
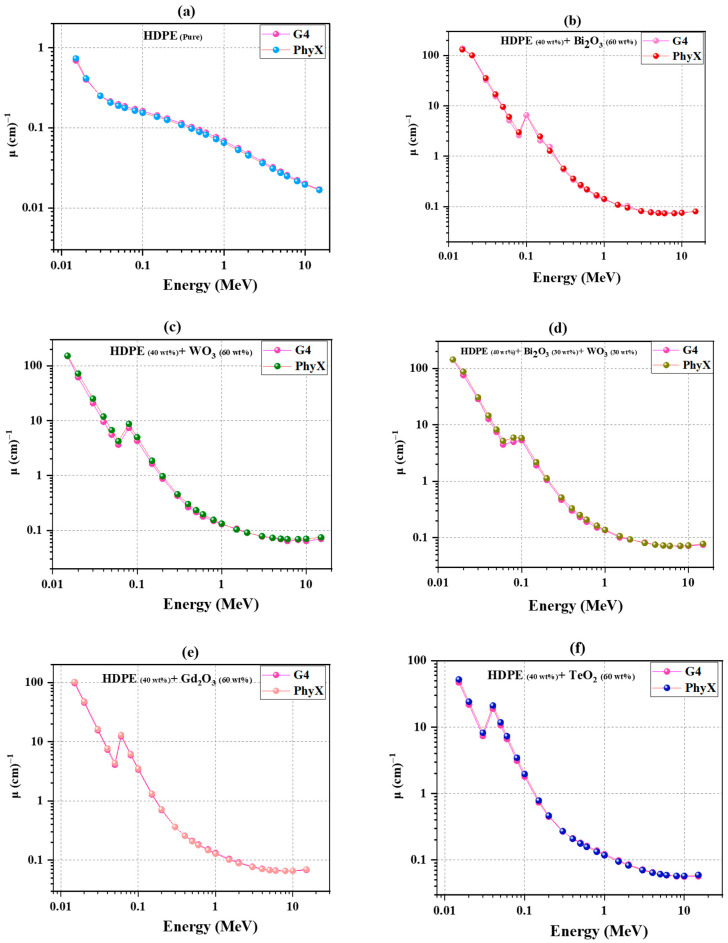
Linear attenuation coefficient (*μ*) versus photon energy (0.015–15 MeV) for HDPE-based composites: (**a**) HDPE; (**b**) HDPE + 60 wt.% Bi_2_O_3_; (**c**) HDPE + 60 wt.% WO_3_; (**d**) HDPE + 30 wt.% Bi_2_O_3_ + 30 wt.% WO_3_; (**e**) HDPE + 60 wt.% Gd_2_O_3_; (**f**) HDPE + 60 wt.% TeO_2_, obtained using Geant4 and Phy-X/PSD.

Figure 2 presents the variation in *μ* with photon energy (0.015–15 MeV) for the HDPE-based composites (C1–C6, as defined in Table 1). The Phy-X/PSD and Geant4 results exhibit very close agreement across the entire photon-energy range, confirming the reliability of both computational approaches. For C1 (HDPE), *μ* decreases steadily from ≈0.7 cm^−1^ at 0.015 MeV to ≈0.16 cm^−1^ at 0.1 MeV, then to ≈0.07 cm^−1^ at 1 MeV, and finally ≈0.02 cm^−1^ at 15 MeV. This smooth decline reflects the expected transition from photoelectric absorption at low energies to Compton scattering and pair-production processes at higher energies. Incorporating high-Z oxides produces a marked enhancement of *μ*, particularly in the low-energy region. At 0.015 MeV, *μ* increases to ≈150 cm^−1^ (Geant4) and ≈153 cm^−1^ (Phy-X/PSD) for C4 (WO_3_), ≈142–143 cm^−1^ for C3 (Bi_2_O_3_/WO_3_), ≈135 cm^−1^ for C2 (Bi_2_O_3_), ≈100 cm^−1^ for C5 (Gd_2_O_3_), and ≈50 cm^−1^ for C6 (TeO_2_), a dramatic improvement compared with neat HDPE (≈0.7 cm^−1^). Distinct discontinuities between 30 and 90 keV correspond to the K-absorption edges of Te, Gd, W, and Bi, accurately resolved by both methods. The pronounced enhancement in this low-energy region reflects the steep *Z^n^* dependence (*n* ≈ 4–5) of the photoelectric effect, which strongly favors composites containing high-Z fillers such as Bi_2_O_3_ and WO_3_. Although Bi_2_O_3_ contains a higher atomic number element than WO_3_, the attenuation performance of HDPE-based composites depends on several factors beyond atomic number alone. In the present composites, the WO_3_-filled system exhibits comparable or slightly higher attenuation due to the combined effects of composite density and energy-dependent photon interaction mechanisms, particularly in the low-energy region where the photoelectric effect dominates. At intermediate and high photon energies, the attenuation performance of WO_3_- and Bi_2_O_3_-filled HDPE becomes very similar, indicating that density-weighted interaction probabilities, rather than atomic number alone, govern the overall shielding behavior.

At 1 MeV, *μ* values cluster around ≈0.13 cm^−1^ for C2–C4, ≈0.12 cm^−1^ for C5–C6, and ≈0.07 cm^−1^ for HDPE, confirming that the oxide-filled systems retain a clear attenuation advantage in the Compton-dominant region. At 10–15 MeV, *μ* remains ≈ 0.07–0.09 cm^−1^ for the composites versus ≈ 0.02 cm^−1^ for HDPE, indicating that dense fillers continue to enhance pair-production interactions at high photon energies. This sustained high-energy enhancement is linked to the increasing probability of electron–positron pair production, whose cross section grows with photon energy and is further amplified by the higher density of oxide-filled composites.

Overall, C4 (WO_3_) exhibits the highest *μ* across nearly all energies, followed by C3 (Bi_2_O_3_/WO_3_) and C2 (Bi_2_O_3_), while C5 (Gd_2_O_3_) and C6 (TeO_2_) show moderate enhancement, and C1 (HDPE) remains the least attenuating.

### 4.2. Mass Attenuation Coefficient (μ/ρ)

**Figure 3:**
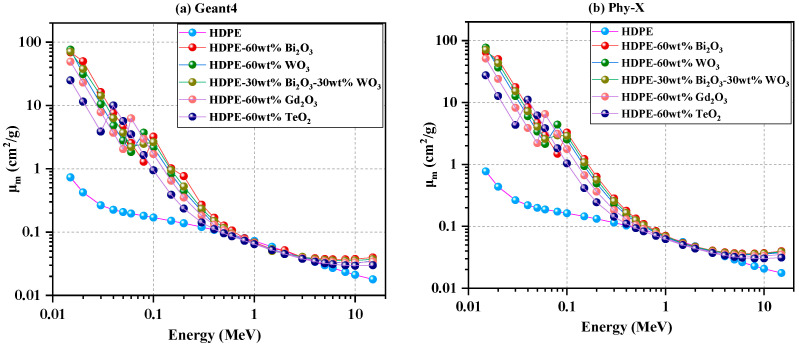
Mass attenuation coefficient (*μ*/*ρ*) of HDPE and oxide-filled HDPE composites as a function of photon energy: (**a**) Geant4 results and (**b**) Phy-X/PSD calculations.

To enable direct comparison between the two computational frameworks, *μ*/*ρ* is plotted using Geant4 (Figure 3a) and Phy-X/PSD (Figure 3b). Both methods display trends consistent with those observed for *μ* in Figure 2.

At 0.015 MeV, the Bi_2_O_3_-, Bi_2_O_3_/WO_3_-, and WO_3_-reinforced composites (C2–C4) record *μ*/*ρ* values of about 70 cm^2^ g^−1^, with WO_3_ reaching approximately 80 cm^2^ g^−1^. Gd_2_O_3_ (C5) and TeO_2_ (C6) exhibit smaller values of roughly 50 cm^2^ g^−1^ and 25 cm^2^ g^−1^, respectively, compared with ≈0.7 cm^2^ g^−1^ for neat HDPE (C1). These elevated coefficients highlight the dominance of the photoelectric effect at low photon energies in materials containing high-atomic-number oxides. The strong low-energy enhancement observed here is fully consistent with the photon-interaction mechanisms discussed in Section 4.1, particularly the steep *Z^n^* dependence (*n* ≈ 4–5) of the photoelectric cross section, which strongly favors materials incorporating heavy elements.

As energy increases toward 0.5–1 MeV, *μ*/*ρ* decreases sharply, and all curves converge to ≈0.07–0.10 cm^2^ g^−1^, signifying the Compton-scattering region where attenuation becomes nearly independent of atomic number. This convergence reflects the fact that Compton interactions depend primarily on electron density rather than Z, reducing compositional differences among the materials.

At 10–15 MeV, *μ*/*ρ* rises slightly to ≈0.03–0.04 cm^2^ g^−1^ for the composites and remains ≈0.02 cm^2^ g^−1^ for HDPE, reflecting renewed pair-production contributions at high photon energies. This divergence at multi-MeV energies follows the same density-driven pair-production trends outlined in Section 4.1, where dense oxide fillers provide more nuclei capable of facilitating electron–positron creation as photon energy increases.

Among all systems, WO_3_ (C4) and Bi_2_O_3_/WO_3_ (C3) show the highest *μ*/*ρ* values throughout the studied energy range, confirming their superior photon-interaction capability within the Geant4 framework.

### 4.3. Mean Free Path (MFP) and Half-Value Layer (HVL)

**Figure 4:**
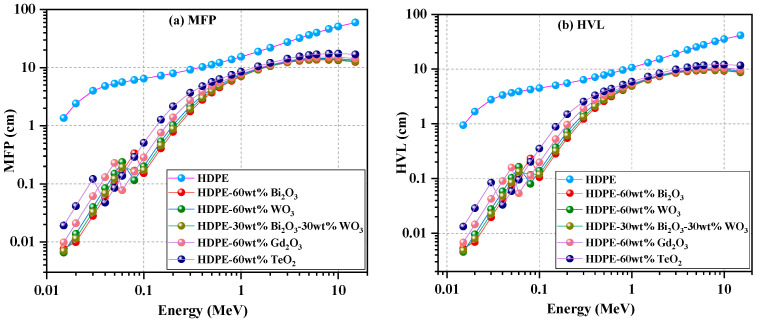
Mean free path (MFP) (**a**) and half-value layer (HVL) (**b**) as functions of photon energy for pure HDPE and HDPE composites reinforced with Bi_2_O_3_, WO_3_, Gd_2_O_3_, TeO_2_, and the Bi_2_O_3_–WO_3_ hybrid formulation.

Figure 4 presents the mean free path (MFP) in panel (a) and the half-value layer (HVL) in panel (b), enabling direct comparison of the photon-attenuation behavior across the studied energy range.

As shown in Figure 4a, at low photon energies below 0.05 MeV, the oxide-reinforced systems exhibit a substantial reduction in photon penetration depth. The Bi_2_O_3_-, Bi_2_O_3_/WO_3_-, and WO_3_-based composites (C2–C4) exhibit MFPs of ≈0.01–0.15 cm, while Gd_2_O_3_ (C5) and TeO_2_ (C6) record slightly higher values of ≈0.1–0.3 cm, compared with ≈1.4–5 cm for neat HDPE (C1). This strong suppression reflects enhanced photoelectric absorption caused by the dense, high-Z oxide filler

As photon energy increases into the Compton region (≈0.1–1 MeV), MFP increases gradually for all materials. Around 0.3 MeV, HDPE reaches about 9 cm, whereas the composites remain between ≈2 and 4 cm, confirming the sustained attenuation advantage of the filled systems. Near 1 MeV, the composites converge around ≈7–8 cm, while HDPE extends to ≈15 cm, indicating that heavy oxide reinforcement continues to reduce photon penetration even under scattering-dominated conditions.

At higher energies above 1 MeV, particularly in the pair-production region (5–15 MeV), all samples show a further smooth rise in MFP. Nevertheless, the composites maintain shorter paths—≈13–15 cm compared with ≈50–60 cm for HDPE—demonstrating that dense oxides preserve significant interaction probability even at high photon energies.

The HVL trends in Figure 4b mirror those of the MFP, as expected from its inverse relationship with the linear attenuation coefficient. At photon energies below 0.05 MeV, the Bi_2_O_3_-, Bi_2_O_3_/WO_3_-, and WO_3_-reinforced composites show HVL values of ≈0.005–0.15 cm, while Gd_2_O_3_ and TeO_2_ fall within ≈0.1–0.3 cm, compared with ≈1–4 cm for HDPE. This confirms the strong photon-absorption capability imparted by dense oxide fillers.

In the intermediate energy region (0.1–1 MeV), HVL increases gradually for all materials. Around 0.3 MeV, HDPE reaches about 6–7 cm, while the composites remain within ≈1–3 cm, confirming the continued effectiveness of the heavy-oxide fillers under Compton-scattering conditions. Near 1 MeV, the filled systems converge around ≈5 cm, whereas HDPE exceeds 10 cm, maintaining a clear attenuation advantage.

At higher photon energies (5–15 MeV), HVL rises gradually but remains markedly lower for the composites, typically ≈9–10 cm compared with ≈25–40 cm for HDPE. This persistent difference indicates that the reinforced materials retain superior shielding performance even in the pair-production processes dominate regime

From a practical shielding perspective, the reduced MFP and HVL values observed for the oxide-reinforced HDPE composites have direct implications for shield thickness optimization. Lower HVL and MFP values indicate that a smaller material thickness is required to attenuate the incident photon intensity by 50%, enabling more compact shielding designs compared with neat HDPE. Similarly, the shorter MFP values reflect a higher probability of photon interaction per unit thickness, which contributes to improved attenuation efficiency within thinner layers. These characteristics are particularly important for applications where weight and space limitations are critical, such as medical radiation protection, portable shielding systems, and aerospace structures. The results, therefore suggest that incorporating high-Z oxides into HDPE can significantly reduce the required shield thickness while maintaining effective radiation attenuation, offering clear advantages over unfilled polymer matrices.

### 4.4. Effective Atomic Number (Z_eff_) and Effective Electron Density (N_eff_)

**Figure 5:**
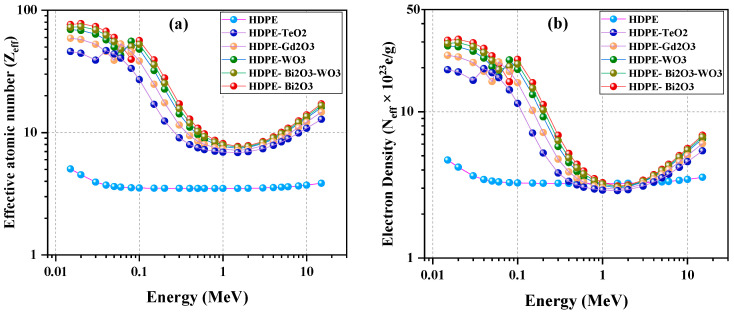
Effective atomic number *Z*_eff_ (panel (**a**)) and effective electron density *N*_eff_ (panel (**b**)) as functions of photon energy from 0.015 to 15 MeV for pure HDPE and HDPE composites containing 60 wt.% oxide fillers (Bi_2_O_3_, WO_3_, Bi_2_O_3_/WO_3_ (30/30 wt.%), Gd_2_O_3_, and TeO_2_) calculated using the Phy-X/PSD database.

Z_eff_ and N_eff_, defined in Section 3.2, describe the composite’s effective atomic number and electron concentration and vary strongly with photon energy. Figure 5a shows the variation in *Z*_eff_, and Figure 5b presents *N*_eff_ for the HDPE-based composites (C1–C6). The addition of heavy oxides markedly enhances both parameters at low photon energies, dominated by the photoelectric effect.

At 0.015 MeV, C1 (HDPE) records approximately *Z*_eff_ ≈ 5.0 and *N*_eff_ ≈ 5 × 10^23^ e g^−1^, while C2 (Bi_2_O_3_) reaches about 80 and 30 × 10^23^ e g^−1^, respectively. Comparable low energy enhancements observed for C3 (Bi_2_O_3_/WO_3_) and C4 (WO_3_) with *Z*_eff_ ≈ 70–73, and *N*_eff_ ≈ 28–29 × 10^23^ e g^−1^, followed by C5 (Gd_2_O_3_) and C6 (TeO_2_), which still exceed the polymer baseline with *Z*_eff_ ≈ 60, and 45 and *N*_eff_ ≈ 24 × 10^23^ e g^−1^ and 20 × 10^23^ e g^−1^, respectively. With increasing energy, above about 0.2 MeV, both *Z*_eff_ and *N*_eff_ decrease and gradually converge among the composites as Compton scattering and pair production become dominant. Around 1 MeV, C2 retains the highest interaction probability (*Z*_eff_ about 8; *N*_eff_ about 3.0 × 10^23^ e g^−1^), while HDPE remains the lowest (*Z*_eff_ ≈ 3–4). However, in this Compton-dominant region, the *N*_eff_ values of all systems—including neat HDPE—become nearly identical, reflecting the fact that Compton scattering depends mainly on the number of electrons per gram rather than on atomic number. At higher energies (up to 15 MeV), minor differences persist, confirming the sustained advantage of oxide-reinforced systems over the neat polymer.

### 4.5. Fast-Neutron Shielding Performance (Σ_R_)

**Figure 6:**
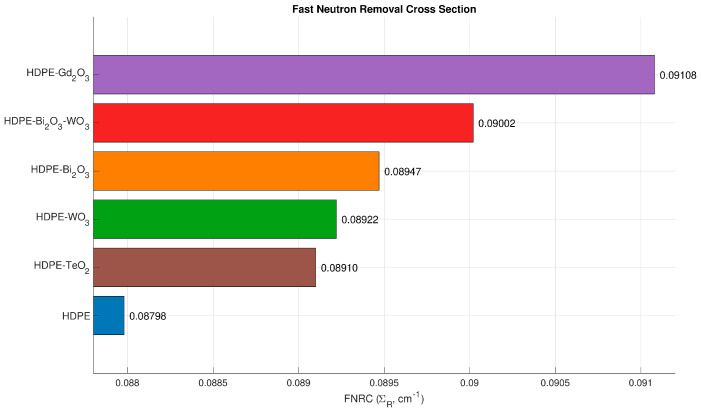
Fast-neutron removal cross section (Σ_R_) for HDPE (C1) and oxide-reinforced composites C2–C6, including Bi_2_O_3_-, Bi_2_O_3_–WO_3_-, WO_3_-, Gd_2_O_3_-, and TeO_2_-filled HDPE.

Figure 6 presents the fast-neutron removal cross section (Σ_R_) for the HDPE-based systems C1–C6. The unfilled polymer (C1) shows the lowest value, Σ_R_ ≈ 0.0879 cm^−1^. All oxide-reinforced composites show a modest but consistent enhancement in Σ_R_, as described by the definition and interpretation provided in Section 3.2. Among the filled systems, C5 (Gd_2_O_3_) achieves the highest Σ_R_ (≈0.0911 cm^−1^), followed closely by the hybrid composite C3 (Bi_2_O_3_–WO_3_) at ≈0.0900 cm^−1^. This superior performance of the Gd_2_O_3_-filled composite is directly linked to the exceptionally large thermal-neutron absorption cross section of gadolinium (≈49,000 barns for natural Gd), one of the highest of any naturally occurring element, which significantly enhances its neutron-capture capability [33]. The Bi_2_O_3_ (C2), WO_3_ (C4), and TeO_2_ (C6) systems yield Σ_R_ values of ≈0.0895, 0.0892, and 0.0891 cm^−1^, respectively, all higher than that of neat HDPE. Although the numerical differences appear minor, the enhancement is physically meaningful, as even small increases in Σ_R_ reflect improved probabilities of fast-neutron attenuation within the composites. This improvement is primarily attributed to the synergistic moderation of hydrogen in HDPE and the superior neutron-capture potential of Gd_2_O_3_, which provides the most notable gain among all examined fillers.

### 4.6. Buildup Factors: Exposure (EBF) and Energy-Absorption (EABF)

EBF and EABF, described in Section 3.2, quantify the contribution of scattered photons to exposure and absorbed dose. Reducing these buildup factors is critical because excessive photon scatter increases dose beyond what primary-beam attenuation predicts, degrading shielding performance and potentially compromising radiation protection requirements.

**Figure 7:**
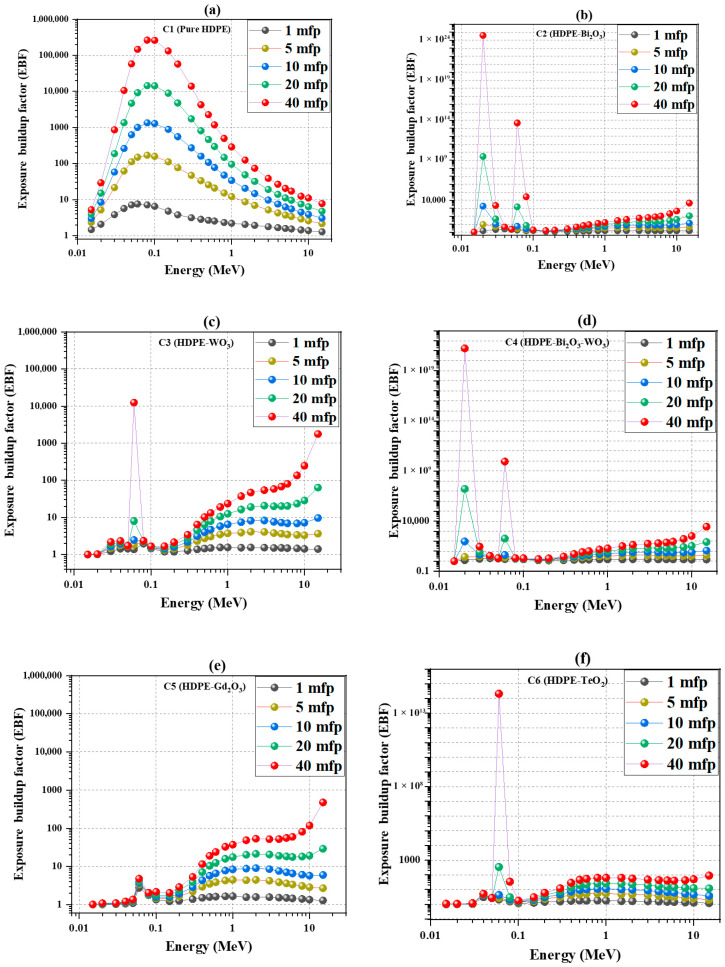
Exposure buildup factor (EBF) as a function of photon energy for different mean free paths (MFPs): (**a**) HDPE; (**b**) HDPE + 60 wt.% Bi_2_O_3_; (**c**) HDPE + 60 wt.% WO_3_; (**d**) HDPE + 30 wt.% Bi_2_O_3_ + 30 wt.% WO_3_; (**e**) HDPE + 60 wt.% Gd_2_O_3_; (**f**) HDPE + 60 wt.% TeO_2_.

Figure 7 illustrates the variation in the exposure buildup factor (EBF) with mean free path (MFP) for HDPE and its oxide-reinforced composites. Under identical conditions, C2 (Bi_2_O_3_) exhibits the lowest buildup, maintaining EBF ≈ 2 even at 40 mean free paths in the low-to-intermediate energy range (approximately 0.05–0.2 MeV). C3 (Bi_2_O_3_–WO_3_) and C4 (WO_3_) show similar behavior, while C5 (Gd_2_O_3_) and C6 (TeO_2_) record slightly higher values but remain far below the polymer baseline (C1). In the Compton-dominated region (≈0.3–1 MeV), the contrast remains pronounced: at 1 MeV and 40 MFP, C1 reaches ≈300, whereas C2–C4 range between 25 and 40 and C5–C6 between 60 and 100. At higher energies (>3 MeV), all composites exhibit a moderate increase in buildup with increasing penetration depth, resulting from secondary photon and positron cascades. At 15 MeV and 40 MFP, C2 and C3 reach ≈(3–4) × 10^3^, whereas C1 remains near 10, highlighting the role of dense oxides in enhancing high-energy photon interaction and attenuation.

**Figure 8:**
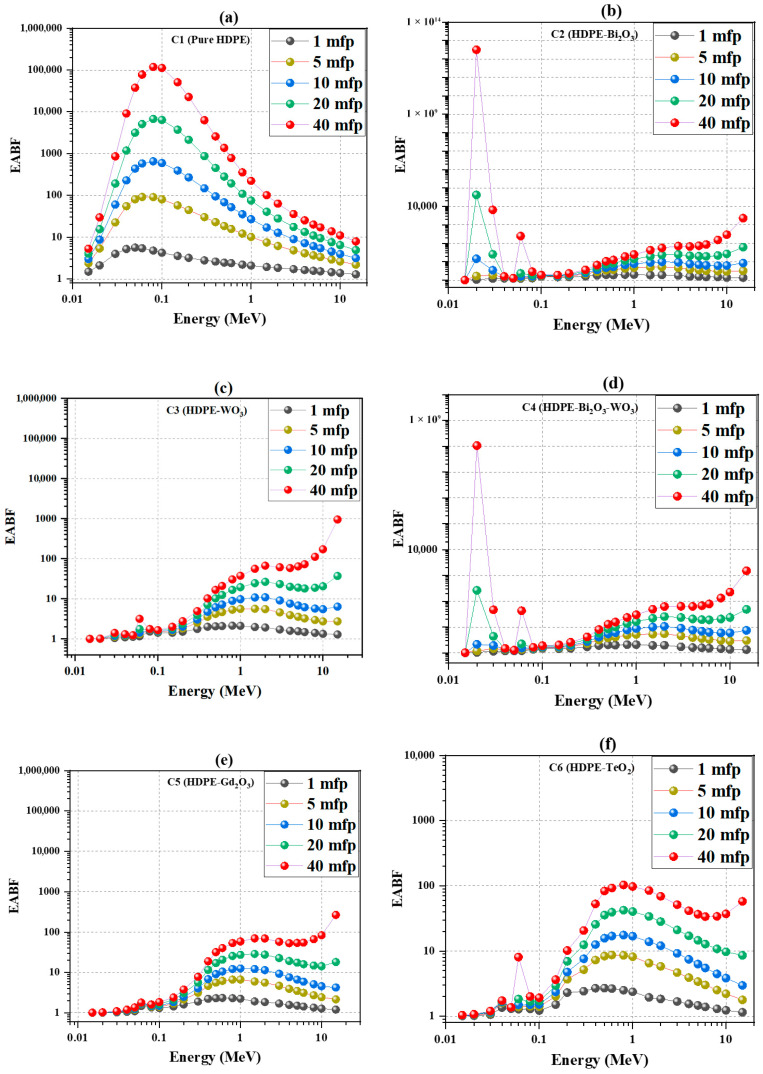
Energy-absorption buildup factor (EABF) as a function of photon energy for different mean free paths (MFPs): (**a**) HDPE; (**b**) HDPE + 60 wt.% Bi_2_O_3_; (**c**) HDPE + 60 wt.% WO_3_; (**d**) HDPE + 30 wt.% Bi_2_O_3_ + 30 wt.% WO_3_; (**e**) HDPE + 60 wt.% Gd_2_O_3_; (**f**) HDPE + 60 wt.% TeO_2_.

Figure 8 shows the energy-absorption buildup factor (EABF) versus MFP. At 0.1 MeV, C1 increases rapidly—from ≈4 at 1 MFP to ≈80 at 5 MFP, ≈600 at 10 MFP, ≈6000 at 20 MFP, and ≈1 × 10^4^ at 40 MFP—while oxide-filled systems remain nearly constant near unity. Across most energies, the composites retain EABF ≈ 2 at both 10 and 40 MFP, confirming strong suppression of secondary photon buildup. The narrow spikes appearing at a few low-energy points arise near the K- or L-absorption edges of the high-Z fillers, where the photon cross sections exhibit abrupt discontinuities. Numerical interpolation across these discontinuities can introduce small overshoots that are not physical interaction processes but artifacts of the tabulated cross-section data, as noted in standard databases such as NIST XCOM [28].

At 1 MeV, C1 reaches ≈200, whereas C2–C4 remain within 20–40, and C5–C6 between 60 and 100. At 15 MeV, the EABF of all systems increases slightly, with the dense composites showing a higher buildup than HDPE, supporting the concept of layered shielding designs that combine hydrogen-rich moderation with high-Z absorption for improved protection against mixed and high-energy radiation fields.

For practical relevance, it is useful to compare the present HDPE–oxide composites with standard commercial shielding materials. Lead provides excellent gamma attenuation because of its very high density (11.34 g·cm^−3^), but its toxicity and rigidity limit its use in many environments. Borated polyethylene (commonly 5–30 wt.% B) is highly effective for neutron moderation yet insufficient for gamma shielding. Barite-concrete offers a non-toxic alternative with moderate gamma attenuation, but is significantly heavier and less mechanically versatile. In contrast, the HDPE-based composites studied here combine effective photon attenuation, enhanced neutron interaction (notably for Gd_2_O_3_-filled systems), mechanical robustness, and reduced weight, underscoring their suitability as practical, lead-free shielding materials for medical, industrial, and aerospace applications.

## 5. Conclusions

This study comprehensively evaluated HDPE composites reinforced with high-Z oxides, Bi_2_O_3_, WO_3_, Gd_2_O_3_, TeO_2_, and a Bi_2_O_3_/WO_3_ hybrid (30/30 wt.%), for efficient gamma-ray and fast-neutron shielding over 0.015–15 MeV using Phy-X/PSD and Geant4 simulations. Incorporating dense oxides produced substantial gains in photon attenuation, particularly at low energies, where WO_3_ and the Bi_2_O_3_–WO_3_ hybrid exhibited the highest linear-attenuation coefficients, followed by Bi_2_O_3_; Gd_2_O_3_ and TeO_2_ provided moderate improvements. Around 0.662 MeV, which corresponds to the gamma-ray energy of the widely used Cs-137 emission line frequently employed in shielding experiments due to its availability as a stable sealed source and its intermediate penetration depth that represents many medical, industrial, and environmental gamma-ray fields, all composites maintained superior attenuation compared with neat HDPE, while Bi- and W-rich systems remained dominant. At multi-MeV energies, attenuation coefficients converged but continued to favor oxide-reinforced systems due to pair-production contributions. On a mass basis, the improvement was most pronounced at low energies, slightly reduced near 1 MeV, and increased again at multi-MeV levels due to pair-production effects. The buildup factors (EBF and EABF) stayed near unity up to ~1 MeV, confirming effective suppression of scattered-photon accumulation. At higher energies, a moderate rise in buildup indicated that layered configurations combining hydrogen-rich moderation and dense absorbers can further optimize performance. The fast-neutron removal cross section (Σ_R_) improved slightly for all filled composites, with Gd_2_O_3_ achieving the highest enhancement, confirming its dual gamma- and neutron-shielding potential. Excellent consistency between Phy-X/PSD and Geant4 outcomes validates the reliability of the hybrid computational framework. These lead-free HDPE-based composites present lightweight, flexible, and environmentally safe alternatives for radiation protection in medical, nuclear, and aerospace applications. Future work should emphasize experimental fabrication with improved filler dispersion, evaluation of mechanical and thermal stability, optimization of multilayer architectures, and benchmarking against conventional shielding materials under realistic radiation conditions.

## Figures and Tables

**Table 1:** Composition and densities of the investigated HDPE-based composites.

ID	Composite	Density (g/cm^3^)	Formula Representation
C1	HDPE	≈0.95	C_2_H_4_
C2	HDPE + 60% Bi_2_O_3_	≈2.01	0.40 C_2_H_4_ + 0.60 Bi_2_O_3_
C3	HDPE + 30% Bi_2_O_3_ + 30% WO_3_	≈2.01	0.40 C_2_H_4_ + 0.30 Bi_2_O_3_ + 0.30 WO_3_
C4	HDPE + 60% WO_3_	≈1.98	0.40 C_2_H_4_ + 0.60 WO_3_
C5	HDPE + 60% Gd_2_O_3_	≈1.99	0.40 C_2_H_4_ + 0.60 Gd_2_O_3_
C6	HDPE + 60% TeO_2_	≈1.90	0.40 C_2_H_4_ + 0.60 TeO_2_

## Data Availability

The data presented in this study were generated through numerical simulations using the Phy-X/PSD and Geant4 codes. All datasets supporting the findings are available from the corresponding author upon reasonable request.
